# The clinical manifestations and treatment outcomes of Behçet's disease: A single‐center experience

**DOI:** 10.1002/hsr2.2238

**Published:** 2024-07-24

**Authors:** Karokh F. Hama Hussein, Hiwa O. Abdullah, Shaho F. Ahmed, Aso N. Qadir, Hoshmand R. Asaad, Fattah H. Fattah, Bnar J. Hama Amin, Dana T. Gharib, Shvan H. Mohammed, Rawezh Q. Salih, Berun A. Abdalla, Fahmi H. Kakamad, Shorsh A. Mohammed

**Affiliations:** ^1^ Smart Health Tower Sulaimani Iraq; ^2^ Department of Gastroenterology Kurdistan Center for Gastroenterology and Hepatology Sulaimani Iraq; ^3^ Kscien Organization Sulaimani Iraq; ^4^ College of Medicine University of Sulaimani Sulaimani Iraq

**Keywords:** autoimmune disease, Behcet disease, Chronic disease, inflammatory disorder

## Abstract

**Background and Aims:**

Behçet's disease is a chronic, multisystemic, and relapsing inflammatory disorder. It lacks a permanent cure, the focus of treatment is on mitigating symptoms, decreasing the frequency and severity of relapses, and preventing life‐threatening complications. This study aims to report the experience of a single center in managing patients with Behçet's disease and discuss the treatment outcomes.

**Methods:**

This study was a retrospective case series conducted over 2 years. All cases were clinically diagnosed according to the International Criteria for Behçet's Disease. The extracted data were demographics, family history, clinical findings, criteria scores, treatment, and outcomes.

**Results:**

A total of 31 patients were included, consisting of 13 males (42%) and 18 females (58%). Most cases were over the age of 30, and both genders were nearly equally distributed among age groups. The most commonly affected site was the oral cavity, observed in 96.77% of cases. Genital, cutaneous, and vascular involvements were more common in males, while females were more likely to have oral, ocular, and musculoskeletal involvements. For various treatment regimens, oral, cutaneous, vascular, and musculoskeletal involvements showed complete response in all cases. Among cases with genital involvement, complete response was achieved in seven cases (41.2%), while four cases (23.5%) showed only partial response, and six cases (35.3%) experienced recurrence. In cases with ocular involvement, only partial responses were observed.

**Conclusion:**

Oral, cutaneous, vascular, and musculoskeletal involvements may have a higher likelihood of a complete response to treatment regimens. However, genital involvement may be the most recurrent manifestation, followed by ocular involvement.

## Key points

The incidence of the disease varies widely across different regions. The risk factors include family history, exposure to environmental factors, and genetic predisposition. Its diagnosis is challenging because the symptoms are not always concomitant and there are no specific diagnostic tests. The focus of treatment is on mitigating symptoms, decreasing the frequency and severity of relapses, and preventing life‐threatening complications.

## 1 INTRODUCTION

Behçet's disease (BD) is a chronic, multisystemic, and relapsing inflammatory disorder characterized by recurrent oral (OUs) and genital (GUs) ulcers, skin lesions, and uveitis.^1^ The incidence of BD varies widely across different regions, with the worldwide prevalence ranging between 1 and 370 cases per 100,000 individuals. It is higher in countries along the ancient Silk Road, including Turkey, Iran, and Japan.^2^, ^3^, ^4^ Risk factors for BD include family history, exposure to environmental factors, and genetic predisposition.^4^ The disease is characterized by an imbalance of T helper 1 (Th1) and T helper 17 (Th17) cells, resulting in a pro‐inflammatory state that leads to tissue damage and multiorgan involvement.^5^ It affects both sexes, but its tendency is not well determined.^6^, ^7^ The disease is relatively uncommon, and the underlying factors are not well understood.^6^, ^8^ The BD diagnosis is challenging, especially when the symptoms are not always concomitant and there is a lack of specific diagnostic tests. However, early diagnosis is critical to prevent serious complications.^7^ As the disease lacks a permanent cure, the focus of treatment is on mitigating symptoms, resolving inflammation, preventing further tissue damage, decreasing the frequency and severity of relapses, and preventing potential life‐threatening complications.^7^

This study aims to report the experience of a single center in managing patients with BD and discuss the treatment outcomes.

## 2 METHOD

### 2.1 Study design

This study was a single‐center retrospective case series conducted over 2 years from November 2020 to December 2022. The ethical approval of the study was obtained from the ethics committee of the University of Sulaimani. The study has been written according to PROCESS guidelines.^9^

### 2.2 Settings

The patients were recruited from the Rheumatology Clinic at Smart Health Tower in Sulaimani. All cases were clinically diagnosed according to the International Criteria for Behçet's Disease (ICBD).^10^ The required data, including demographics, family history, clinical findings, criteria scores, treatment, and outcomes, were extracted from patients' medical records in the clinic's database after de‐identifying patient data.

### 2.3 Inclusion criteria

The study included all cases of BD according to the ICBD criteria that were treated between November 2020 and December 2022.

### 2.4 Exclusion criteria

All patients with the following properties were excluded; outpatient cases who previously received treatment for BD, patients with other chronic diseases, and seasonal allergies.

### 2.5 Data collection and analysis

The data were organized using Microsoft Excel 2019. As the study lacks any quantitative data synthesis, the qualitative analysis (descriptive statistics) was performed using the Statistical Package for the Social Sciences (SPSS) software (version 25). The data were presented as mean, standard deviation, frequencies, and percentages.

## 3 RESULTS

A total of 31 patients were included in this study, consisting of 13 males (42%) and 18 females (58%). The age range was between 9 and 65 years, with a mean age of 38 years ±4.08 (35.92 ± 4.07 for males and 39.61 ± 6.29 for females). Most cases were over the age of 30, and both genders were nearly equally distributed among age groups. Family history was positive for the disease in eight cases (25.8%). C‐reactive protein levels were high, above the normal limit of 5 mg/dL, in both genders. The most commonly affected site was the oral cavity, observed in 96.77% of cases. Genital and ocular involvements were also common, with a prevalence of 54.84% for each. Genital, cutaneous, and vascular involvements were more common in males, while females were more likely to have oral, ocular, and musculoskeletal involvements (Table 1). Based on the ICBD criteria, disease scores were primarily 3 (51.6%) and 4 (29%), with only one case reaching a score of 7 (3.2%). The majority of female cases (66.7%) had 3 scores, whereas male cases were mostly distributed between scores of 3 (30.8%) and 4 (30.8%). The treatment included prednisolone and colchicine (100%), azathioprine (Imuran) (74.2%), mometasone furoate (Elica) (45.2%), and infliximab (6.5%). For various treatment regimens, oral, cutaneous, vascular, and musculoskeletal involvements showed complete response in all cases. Among cases with genital involvement, complete response was achieved in seven cases (41.2%) with various treatment combinations, while four cases (23.5%) showed only partial response, and six cases (35.3%) experienced recurrence. In cases with ocular involvement, only partial responses were observed (Table 2). Details about the included cases are summarized in Table 3.

**Table 1:** Demographic and clinical characteristics of patients according to gender.

Characteristics	Patients (*n* = 31)	Male (*n* = 13)	Female (*n* = 18)
Age	31 (100%)	35.92 ± 4.07	39.61 ± 6.29
<20	1 (3.2%)	0 (0%)	1 (5.6%)
20–29	5 (16.1%)	3 (23.1%)	2 (11.1%)
30–39	14 (45.2%)	6 (46.2%)	8 (44.4%)
>40	11 (35.5%)	4 (30.8%)	7 (38.9%)
Fhx	8 (25.8%)	3 (23.1%)	5 (27.8%)
CRP	31	19.54 (6.44)	19.17 (6.35)
ESR	31	5.46 (6.09)	7.06 (7.67)
Involved sites			
Oral	30 (96.77%)	12 (92.3%)	18 (100%)
Genital	17 (54.84%)	11 (84.6%)	6 (33.3%)
Skin	14 (45.2%)	6 (46.2%)	8 (44.4%)
Eye	17 (54.84%)	6 (46.2%)	11 (61.1%)
VASC	9 (29%)	4 (38.5%)	5 (22.2%)
MSK	14 (45.2%)	4 (30.8%)	10 (55.6%)
Criteria			
3	16 (51.6%)	4 (30.8%)	12 (66.7%)
4	9 (29%)	4 (30.8%)	5 (27.8%)
5	2 (6.45%)	2 (15.4%)	0 (0%)
6	3 (9.68%)	2 (15.4%)	1 (5.6%)
7	1 (3.2%)	1 (7.7%)	0 (0%)
Medication use			
Prednisolone	31 (100%)	13 (100%)	18 (100%)
Colchicine	31 (100%)	13 (100%)	18 (100%)
Elica	14 (45.2%)	6 (46.2%)	8 (44.4%)
Imuran	23 (74.2%)	9 (69.2%)	14 (77.8%)
Infliximab	2 (6.5%)	2 (15.4%)	0 (0%)

Abbreviations: CRP, C‐reactive protein; ESR, erythrocyte sedimentation rate; Fhx, family history; MSK, musculoskeletal; VASC, vascular.

**Table 2:** Treatment approaches and success rates according to the involved sites.

Drug combinations	Treatment response (complete/partial/recurrent)					
	Oral (*n* = 30)	Genital (*n* = 17)	Skin (*n* = 14)	Eye (*n* = 17)	VASC (*n* = 9)	MSK (*n* = 14)
Pr.Co. (*n* = 5)	5/0/0	2/1/2				1/0/0
Pr.Co.iM. (*n* = 10)	10/0/0	1/1/1		0/9/0	3/0/0	3/0/0
Pr.Co.iM.iN. (*n* = 2)	1/0/0	0/2/0		0/2/0	1/0/0	1/0/0
Pr.Co.E. (*n* = 4)	4/0/0	2/0/2	4/0/0			1/0/0
Pr.Co.E.iM. (*n* = 10)	10/0/0	2/0/1	10/0/0	0/6/0	5/0/0	8/0/0

Abbreviations: Co., colchicine; E., Elica; iM, Imuran; iN, infliximab; MSK, musculoskeletal; Pr., prednisolone; VASC, vascular.

**Table 3:** Demographic and clinical characteristics of individual cases.

Patient	Gender	Age	Fhx	CRP	ESR	Oral	O.C.	Genital	O.C.	Skin	O.C.	Eye	O.C.	VASC	O.C.	MSK	O.C.	Drug	Criteria
1	M	42	+	25	18	+	C	+	P	−	−	+	P	−	−	−	−	Pr.Co.iM.	5
2	F	53	+	23	15	+	C	−	−	+	C	+	P	−	−	+	C	Pr.Co.E.iM.	4
3	F	63	+	24	20	+	C	+	R	+	C	−	−	−	−	+	C	Pr.Co.E.	4
4	F	65	+	25	25	+	C	+	R	+	C	−	−	−	−	−	−	Pr.Co.E.	4
5	M	26	+	22	20	−	−	+	P	−	−	+	P	−	−	−	−	Pr.Co.iM.iN.	4
6	F	40	+	21	22	+	C	−	−	+	C	+	P	−	−	+	C	Pr.Co.E.iM.	3
7	F	49	+	8	3	+	C	−	−	+	C	+	P	−	−	+	C	Pr.Co.E.iM.	3
8	M	25	+	5	2	+	C	+	P	−	−	+	P	+	C	+	C	Pr.Co.iM.iN.	6
9	F	24	−	6	3	+	C	−	−	−	−	+	P	+	C	−	−	Pr.Co.iM.	4
10	M	37	−	8	3	+	C	+	P	−	−	−	−	−	−	−	−	Pr.Co.	3
11	F	32	−	10	4	+	C	−	−	+	C	−	−	+	C	+	C	Pr.Co.E.iM.	3
12	F	9	−	9	2	+	C	−	‐	−	−	+	P	−	−	−	−	Pr.Co.iM.	3
13	M	24	−	20	4	+	C	+	C	−	−	+	P	+	C	−	−	Pr.Co.iM.	6
14	F	34	−	22	4	+	C	−	−	−	−	+	P	−	−	+	C	Pr.Co.iM.	3
15	M	37	−	18	4	+	C	+	C	−	−	−	−	−	−	+	C	Pr.Co.	3
16	F	36	−	17	2	+	C	+	R	+	C	+	P	−	−	+	C	Pr.Co.E.iM.	6
17	M	33	−	25	2	+	C	+	C	+	C	−	−	−	−	−	−	Pr.Co.E.	4
18	M	38	−	23	3	+	C	+	C	+	C	−	−	+	C	−	−	Pr.Co.E iM.	5
19	F	27	−	24	2	+	C	+	R	−	−	−	−	+	C	+	C	Pr.Co.iM.	4
20	F	37	−	24	2	+	C	−	−	−	−	+	P	−	−	−	−	Pr.Co.iM.	3
21	M	33	−	23	2	+	C	+	C	−	−	−	−	−	−	−	−	Pr.Co.	3
22	F	36	−	25	5	+	C	+	R	−	−	−	−	−	−	−	−	Pr.Co.	3
23	M	36	−	27	2	+	C	−	−	+	C	−	−	+	C	−	−	Pr.Co.E.iM.	3
24	M	47	−	21	5	+	C	+	C	+	C	−	−	−	−	−	−	Pr.Co.E.	4
25	F	46	−	20	5	+	C	−	−	−	−	+	P	−	−	−	−	Pr.Co.iM.	3
26	F	57	−	20	5	+	C	−	−	+	C	−	−	+	C	+	C	Pr.Co.E.iM.	3
27	M	49	−	19	3	+	C	−	−	+	C	+	P	−	−	+	C	Pr.Co.E.iM.	4
28	M	40	−	18	3	+	C	+	C	+	C	+	P	+	C	+	C	Pr.Co.E.iM.	7
29	F	32	−	22	2	+	C	−	−	−	−	+	P	−	−	−	−	Pr.Co.iM.	3
30	F	37	−	22	3	+	C	−	−	−	−	+	P	−	−	+	C	Pr.Co.iM.	3
31	F	36	−	23	3	+	C	+	R	−	−	−	−	−	−	−	−	PC	3

Abbreviations: −, negative; +, positive; C, complete recovery; Co., colchicine; CRP, C‐reactive protein; E., Elica; ESR, erythrocyte sedimentation rate; F, female; Fhx, family history; iM, Imuran; iN, infliximab; M, male; MSK, musculoskeletal; O.C., outcome; P, partial recovery; Pr., prednisolone; R, recurrent; VASC, vascular.

## 4 DISCUSSION

The disease is characterized by recurring episodes of inflammation that can involve multiple organs alongside distinct mucocutaneous manifestations (MCM). In 1937, a dermatologist named Hulusi Behçet initially reported this condition as a triad of recurring OUs, GUs, and uveitis.^11^ The underlying mechanisms responsible for the development of BD are not yet fully understood. The most supported hypothesis is that an aberrant immune response is triggered in genetically susceptible individuals by auto‐antigens or environmental factors.^11^ This disease profoundly impairs the quality of life and can result in substantial economic burdens for both patients and society.^12^

The distinctive clinical features of BD are primarily MCM, including OUs (92%–100%), followed by GUs (57%–93%), and cutaneous vasculitic ulcers (38%–99%). Ocular and joint involvements can also be seen in 29%–100% and 16%–84% of the cases, respectively.^13^ In addition, MCM are the commonly utilized diagnostic criteria for BD due to their high sensitivity and specificity, they comprise 5 out of the 10 scores of the ICBD criteria.^10^ Some patients initially have only MCM, and organ involvement may occur after months or years of symptom onset.^11^

The OUs are painful ulcers that develop on the oral mucosa and typically manifest as erythematous, vesiculopustular lesions that transform into oval or round ulcers within 2 days. These ulcers are characterized by an encircling red halo and a central area with a grayish‐yellow necrotic base. They commonly appear on nonkeratinized mucosal surfaces like the lips, tongue, buccal mucosa, soft palate, and mouth floor.^13^, ^14^ The activity of OUs is a potential predictor of new organ involvement in young males. Despite a decrease in the prevalence and severity of MCM with age, OUs can remain persistent over time.^13^, ^15^ In various populations such as Turkey, Egypt, Greece, Japan, and Italy, OUs have been documented as the initial presentation in approximately 63.4%–88.7% of cases. On average, additional symptoms necessary for diagnosis appear around 4 years after the onset of OUs.^11^

The second most prevalent symptom following OUs is GUs, which are considered the most specific clinical presentation for diagnosis. The presentation of GUs resembles that of OUs but with a lower recurrence rate. GUs are characterized by oval or round lesions lined by a yellowish or necrotic membrane. They may appear as single or multiple lesions and are typically found on the scrotum in males and the labia in females. These lesions can lead to dyspareunia, severe pain, and difficulties in urination and physical activities.^13^, ^14^ Papulopustular lesions, observed in 65%–96% of cases, are the predominant skin manifestations of BD. They are identified by sterile pustules appearing on an erythematous base. These lesions initially appear as papules and progress into pustules within 2 days. The most common sites for these lesions are the face, trunk, and lower extremities.^11^ According to several previous studies, the most common presentations were OUs (100%), GUs (50%–64.8%), skin lesions (85%–95.8%), arthritis (69%), ocular lesion (62%), uveitis (18.3%–54%), and vascular involvement (34.27%).^7^, ^16^, ^17^, ^18^ In the present series, OUs were the most common presentation in almost all cases (96.77%). They appeared in all female cases and 92.3% of male cases. Genital, cutaneous, and vascular involvements were seen in nearly 55%, 45.2%, and 29% of the cases, respectively, with a predilection for males over females. However, in terms of ocular and musculoskeletal involvements, females were more commonly affected than males.

This inflammatory disorder may affect multiple organs or systems such as the eyes, joints, nervous system, vascular system, and gastrointestinal system. It has a high mortality rate, particularly in young males, with significant causes of death being large‐vessel, gastrointestinal, neurological, and cardiac involvements.^11^ The disease commonly manifests during the third to fourth decades of life, however, there are controversies regarding gender distribution.^13^ Fernández‐Ávila et al. reported a female‐to‐male ratio of 2.2:1 among 523 cases of BD, with 68% of the cases being female.^19^ Calambia et al., Davatchi et al., and Miyagawa et al. all found a gender predilection towards females.^7^, ^18^, ^20^ In contrast, two other studies reported a male predilection of 81.25% and 62.5%, respectively.^16^, ^17^ The disease is significantly more prevalent in populations residing across the ancient “Silk Road” with Turkey exhibiting the highest prevalence rates.^13^ Geographical regions show variations in the disease, and discussions continue regarding the role of familial and environmental factors on BD incidence.^11^ In our study, 58% were female, while 42% were male. The mean age for males was 35.92, and for females, it was 39.61. Familial history was positive in 25.8% of the cases.

Due to the absence of a definitive laboratory indicator for the disease, diagnosis primarily relies on clinical symptoms. The ICBD diagnostic criteria have recently emerged as widely utilized criteria. According to these criteria, each episode of OUs, GUs, and ocular involvement is assigned a score of two points, while other symptoms such as skin lesions, vascular involvement, neurological manifestations, and a positive skin pathergy test are given one point. A total score of ≥ 4 indicates a diagnosis of BD, while a score of three points suggests a probable BD diagnosis.^10^, ^11^ In the present study, regarding the ICBD criteria, more than half of the cases achieved a score of three, while only one case reached a score of seven.

The effectiveness of treatment in BD can be influenced by several variables, such as the affected organ, the severity, and duration of involvement, number of inflammatory attacks, age, and gender. The primary goal of treatment should be the prompt suppression of these attacks and the prevention of future ones, especially during the active stages of the disease, to prevent irreversible organ damage.^21^ Because BD is a multisystemic inflammatory disease, the effectiveness of topical treatments is generally restricted to the specific area of application. Therefore, it is advised to combine topical treatments with systemic therapies rather than relying on them alone. However, Alpsoy et al. proposed that in certain cases, topical treatments may be used as standalone options in patients with no major organ involvement who have experienced a prolonged absence of new attacks.^11^

Colchicine serves as an effective systemic treatment for a wide range of MCM and can be considered the initial therapy for both mucocutaneous and articular symptoms. In cases where colchicine alone proves insufficient, the addition of benzathine penicillin injections every 3 weeks can enhance the efficacy of colchicine, particularly on OUs, GUs, erythema nodosum (EN)‐like lesions, and articular symptoms.^21^, ^22^ In cases of acute and severe mucocutaneous attacks, corticosteroids are commonly used due to their rapid anti‐inflammatory effects. They are particularly preferred when major OUs, GUs, or severe OUs and GUs with EN‐like lesions are present. However, their side effect profile restricts their prolonged utilization, and they do not have lasting effects on the prognosis of the disease. In clinical practice, a recommended dosage of prednisolone is 40–60 mg/day for MCM, gradually tapering off and discontinuing within 4–6 weeks. It is worth noting that the use of corticosteroids as a standalone treatment during acute attacks is not efficient.^21^ Tumor necrosis factor (TNF)‐α inhibitors have demonstrated remarkable efficacy in treating BD across a wide range of organ involvements. These inhibitors have a significant role in inhibiting the risk of visual loss in cases of ocular involvement and decreasing mortality rates in cases involving gastrointestinal, neurological, and severe vascular manifestations. Hence, TNF‐α inhibitors should be administered, particularly in young male patients who present with MCM along with significant organ involvement.^23^ Davatchi et al. conducted a comparative analysis of the efficacy of different treatment approaches for BD. Their research revealed that the combined administration of cyclophosphamide and corticosteroids proved to be more effective in managing ocular manifestations of BD than corticosteroids alone.^24^ Others indicated the promising potential of colchicine in treating mucocutaneous and joint‐related symptoms.^20^ In another study, it was demonstrated that the inclusion of Interferon‐α2b (pegylated) in the treatment regimen could reduce the necessity for corticosteroids in BD patients with both ocular and systemic involvement.^25^ Furthermore, when comparing cyclosporine with conventional treatment (prednisolone and azathioprine), cyclosporine emerged as a more effective option for managing various manifestations of BD, including OUs and GUs, superficial thrombophlebitis, cutaneous lesions, as well as articular and neurologic manifestations.^26^ In this series, we treated our cases using various combined regimens, which included prednisolone, colchicine, mometasone furoate (Elica), azathioprine (Imuran), and infliximab. Among different treatment approaches, oral, cutaneous, vascular, and musculoskeletal involvements exhibited complete responses in all cases. However, genital involvement demonstrated the least favorable overall response, with only 7 out of 17 cases achieving a complete response and 6 cases experiencing recurrence. In spite of the fact that cases with three scores are regarded as probable BD, in our study, all of these cases responded to the treatment. This study is disadvantaged through several aspects such as a small sample size, including cases that are considered as probably BD according to ICBD criteria, and a lack of proper statistical analysis. The findings of this study are prone to bias due to the nature of the study design, so further research with a proper design is recommended. To validate our reference list, we have checked all cited papers to exclude predatory publications.^27^

In conclusion, the treatment success of BD can vary based on the manifestation of the disease. Oral, cutaneous, vascular, and musculoskeletal involvements may have a higher likelihood of a complete response to treatment regimens. However, genital involvement may be the most recurrent manifestation, followed by ocular involvement.

## AUTHOR CONTRIBUTIONS

**Karokh F. Hama Hussein**: Investigation; methodology; validation; writing—review and editing. **Hiwa O. Abdullah**: Conceptualization; investigation; writing—original draft; writing—review and editing. **Shaho F. Ahmed**: Data curation; investigation; validation; visualization; writing—review and editing. **Aso N. Qadir**: Investigation; methodology; validation; visualization; writing—review and editing. **Hoshmand R. Asaad**: Conceptualization; data curation; investigation; methodology; resources; validation; writing—review and editing. **Fattah H. Fattah**: Data curation; formal analysis; investigation; methodology; validation; visualization; writing—review and editing. **Bnar J. Hama Amin**: Conceptualization; data curation; methodology; software; validation; writing—review and editing. **Dana T. Gharib**: Conceptualization; formal analysis; investigation; methodology; visualization; writing—review and editing. **Shvan H. Mohammed**: Data curation; investigation; methodology; validation; writing—review and editing. **Rawezh Q. Salih**: Conceptualization; data curation; software; supervision; validation; writing—review and editing. **Berun A. Abdalla**: Investigation; methodology; visualization; writing—original draft; writing—review and editing. **Fahmi H. Kakamad**: Conceptualization; investigation; methodology; project administration; validation; visualization; writing—review and editing. **Shorsh A. Mohammed**: Conceptualization; data curation; writing—review and editing.

## CONFLICT OF INTEREST STATEMENT

The authors declare no conflict of interest.

## ETHICS STATEMENT

Written informed consent was obtained from the patients for publication. The study was approved by the ethics committee of the Sulaimani University.

## TRANSPARENCY STATEMENT

The lead author Fahmi H. Kakamad affirms that this manuscript is an honest, accurate, and transparent account of the study being reported; that no important aspects of the study have been omitted; and that any discrepancies from the study as planned (and, if relevant, registered) have been explained.

## Data Availability

The datasets generated and analyzed during the current study are available from the corresponding author upon reasonable request.
